# NEW BOWEL PREPARATION TECHNIQUE FOR COLONOSCOPY: CLINICAL TRIAL COMPARING AQUANET AND MANNITOL

**DOI:** 10.1590/0102-672020180001e1393

**Published:** 2018-08-16

**Authors:** Roberto Luiz KAISER-JÚNIOR, Luiz Gustavo DE-QUADROS, Mário FLAMINI-JÚNIOR, Mikaell Alexandre Gouvea FARIA, Juan Carlos Ochoa CAMPO, Vera Lúcia DE-OLIVEIRA, Idiberto José ZOTARELLI-FILHO

**Affiliations:** 1 Kaiser Clinic and Hospital, Endoscopy and Colonoscopy, São José do Rio Preto, SP;; 2 Hospital Beneficência Portuguesa, Colonoscopy, São José do Rio Preto, SP; 3 Faculty of Medicine of the ABC, Endoscopy, Santo André, SP;; 4 Faculty of Medicine, Unilago, Colonoscopy, São José do Rio Preto, SP, Brasil

**Keywords:** Colonoscopy, Clinical protocols, Clinical trial, Comparative study, Propensity score, Colonoscopia, Procedimentos clínicos, Estudo clínico, Estudo comparativo, Pontuação de propensão

## Abstract

**Background:**

Fifty-five percent of Americans aged 50-65 are submitted to colonoscopy. For over 65-year, this number increases to 64%. In Brazil, it is forecast that the population submitted to colonoscopy will grow, even though inadequate preparation is still a major problem.

**Aim:**

To analyze the quality of a new intestinal preparation technique, Aquanet EC-2000^®^, compared to oral Mannitol solution.

**Methods:**

This prospective longitudinal study enrolled 200 patients with indication for colonoscopy. The sample was randomly allocated to two groups of 100; one group received Aquanet EC-2000^®^ to prepare for colonoscopy and the other Mannitol solution. The Boston scale was used to analyze the results.

**Results:**

As expected both preparations produced similar results with the bowel cleansing of the different regions of the colon being classified as Boston scale 3 (excellent) in most patients (p>0.05).

**Conclusion:**

The results of bowel preparation using Aquanet EC-2000^®^ were similar to using Mannitol solution.

## RESUMO

**Racional:**

Cinquenta e cinco por cento dos norte-americanos entre 50-65 anos fazem colonoscopia. Acima de 65 anos o número foi de 64%. No Brasil, estima-se crescente aumento da população submetida à colonoscopia, apesar da preparação inadequada ainda ser um grande problema.

**Objetivo:**

Analisar e comparar a qualidade do novo método de preparo intestinal por meio do Aquanet EC-2000^®^ frente ao uso de solução oral de Manitol.

**Método:**

Por randomização 200 pacientes foram divididos em dois grupos de 100. Um recebeu Aquanet EC-2000^®^ e o outro Manitol. O presente estudo seguiu modelo prospectivo longitudinal por meio da seleção de 200 pacientes com indicação à colonoscopia, formando dois grupos de 100. Para analisar os resultados foi utilizada a escala de Boston.

**Resultados:**

Ambos os preparos foram estatisticamente significativos com p<0,05. A escala 3 de Boston foi a mais frequente para ambos os métodos.Além disso, na estatística aplicada às diferentes regiões do cólon para ambos os procedimentos as proporções observadas concordaram com o esperado (3-excelente).

**Conclusão:**

Os resultados do preparo intestinal utilizando Aquanet EC-2000^®^ foram semelhantes aos do Manitol.

## INTRODUCTION

Colonoscopy is currently the gold standard for the investigation of the mucosa of the colon, rectum and terminal ileum, according to randomized multicenter clinical trials related to the detection of colorectal diseases^16^. About 55% of Americans aged 50-65 years had colonoscopy and over 65 the number of exams rose to 64%. These data correspond to about three times more than the 2000 index. As a corollary, the incidence of bowel cancer has dropped by 30% in the last ten years in the United States, according to the American Cancer Society report^7^ ^,^ ^10^ ^,^ ^13^ ^,^ ^22^. In Brazil, in the population submitted to colonoscopy, an increase is estimated on doing it, although inadequate preparation is still a major problem^17^, leading to a repetition of the procedure^16^ ^,^ ^22^.

The quality of colonoscopy is related to the efficacy of its preparation, whether using bowel cleansing devices or oral laxatives^2^ ^,^ ^13^. Inadequate bowel cleansing also has a negative impact on success rates of cecal intubation, prolonging the procedure, decreasing the sensitivity of polyp detection and increasing cost^17^. In addition, efficient cleaning is imperative to identify and treat colorectal cancer, one of the leading causes of death worldwide, with an incidence of 900,000 cases per year^10^ ^,^ ^13^.

Thus, it is necessary to improve the visualization of colonoscopy through better methods of intestinal cleansing^6^ ^,^ ^9^ ^,^ ^23^. The use of Aquanet EC-2000^®^ (AQ) equipment increases the efficiency of retrograde bowel lavage. Water is triple filtered and uses a pressure and gravity system for mechanical removal of fecal contents. The literature has shown that other equipment such as Jetprep (Jetprep Ltd, Herzliya, Israel), Medjet, and ColonoScoPrepTM improve bowel cleansing, are safe, effective and well tolerated by patients^1^ ^,^ ^3^.

Manitol is inexpensive, easy to administer, rapid effect, relative adhesion of the patient and with few side effects, and is as efficient as the other products in use. This result was confirmed by Nahas et al.^14^ in 1,234 colonoscopies with only 15 patients (1.2%) of this group with inadequate cleaning, interfering at the end of the examination.

Despite the good attributions as a laxative, the opportunity for the use of Mannitol in the surgical preparation of the large intestine had a relatively short life, since it was considered to cause an increase in infection of the operative wound, a fact often attributed to the increase in the number of *Escherichia coli* ^9^. In addition, the most important reason for the prohibition of Mannitol was the form of its use in the preparation of the large intestine for the endoscopic examinations that favored the production of combustible gases, due to its fermentation by colonic bacteria^8^ ^,^ ^9^.

As a consequence, comparing the quality of the large intestine gas mixture in patients prepared with Mannitol and in patients prepared with castor oil, it was observed that 60% of the patients prepared with Mannitol had intestinal amounts of hydrogen and potentially explosive methane^8^.

The objective of the present clinical trial was to analyze and compare the intestinal preparation quality score scale between the AQ device and oral solution of Mannitol (M).

## METHODS

### Study design

It is a prospective longitudinal study by means of the selection of 200 patients with indication for colonoscopy, forming two groups of 100. One received the treatment with AQ and the other group M. The first one received dietary guidance and the second one orientation to laxative administration. The study was approved by the Research Ethics Committee of Hospital Beneficência Portuguesa under number 655.036 on May 19, 2014.

### Mannitol method

All 100 participants were instructed to ingest 1 l of 10% Mannitol (500 ml of 20% Mannitol and 500 ml of orange juice) 12 h prior to examination. It was also requested liquid diet the day before.

### **Aquanet EC-2000** ^®^ **Method**

All 100 participants were submitted to retrograde preparation only using AQ for intestinal lavage 1 h prior to examination. The previous day was requested a liquid diet. Patients were placed in left lateral decubitus or dorsal decubitus with flexed limbs and introduced a rectal cannula. It was connected to the AQ through a plastic hose. Only water at 36^o^ C triply filtered with carbon passages, microsediments and ultraviolet light was used. Water infusion was first performed using gravity and then pressure, which increases the efficiency of intestinal preparation. The cleaning process was monitored by the operator until there was clear liquid in the display of the output contents of the equipment. The average time of the entire process reported by the operator was 30 min.

### Classification of the preparation

Classification for both AQ and Mannitol followed the Boston Scale^21^. It was used to evaluate the quality of intestinal cleansing in each of the right, transverse and left (cecum, ascending, transverse including angles, descending, sigmoid and rectum) segments on a 0 to 3 scale. The averages of the three scores were then added to get the final score in a scale of 0 (the minimum value corresponding to unprepared colon) to 9 (maximum value, which corresponds to excellent preparation without any residual trait). The preparation was considered inadequate when the final score was less than 54.

### Participants

Patients were selected from all the ones who needed colonoscopy. Were included those who were between 14-90 years old and who should have had more than three bowel movements per week for a previous month. The following conditions were exclusion criteria: pregnancy (confirmed by pregnancy test), acute abdomen, previous colorectal surgery, hemorrhoids or endoscopic procedures, known intestinal diseases, upper gastrointestinal surgery, uncontrolled angina and/or myocardial infarction in the last three months, heart failure, congestive heart failure, uncontrolled hypertension, renal failure, or known hypersensitivity to the active principles.

### Statistical analysis

All information was compiled in Excell worksheet and then analyzed in the MinitabPro^17^ statistical program. Descriptive statistical measures of frequency, mean, and standard deviation were used in relation to the scores of the Boston Scale. Anderson-Darling normality was tested for subsequent statistical treatment. The Kruskal-Wallis treatment was also performed between each variable of each segment of the colon. The chi-square test (test G (Williams) was also used to analyze polarization between the studied groups. The interference of variables in the primary outcome was analyzed by means of linear regression for continuous variables. For all the tests was adopted alpha level of 0.05. Primary outcome was the percentage of patients classified as “successful” (excellent) according to the Boston Scale^4^, and secondary outcome was the analysis of the influence of continuous or categorical predictors on the quality of bowel preparation.

## RESULTS

Participants’ characteristics regarding age, gender, intestinal habit, constipation and diarrhea are listed in Table 1. Both M and AQ preparations were statistically significant at p>0.05 between each segment of the colon. In addition, the maximum score of 3 for each segment according to the Boston Scale was the most frequent for both methods (Table 2). In relation to group M, the mean values were 2.42 in the right colon, 2.23 in the transverse and 2.10 in the left, giving 6.75 to the final score. Regarding AQ, the mean values were 2.34 in the right colon, 1.64 in the transverse and 2.10 in the left, with a final score of 6.10 (Table 2).

**TABLE 1:** Characteristics of participants in relation to preparation Aquanet and Mannitol

Participant data and type of intestinal preparation	Aquanet (AQ)	Manitol (M)	p
Age (years)	58 (±17)	53 (±16)	<0.05
Gender	94% female	95% female	<0.05
Intestinal habit	90% normal	60% normal	<0.05
Constipation	8%	35%	>0.05
Diarrhea	2%	5%	<0.05

**TABLE 2:** Frequency, score and non-parametric correlation values of each segment of the colon, with p>0.05

BOSTON SCALE SCORE	Rectum - M	Frequency	Rectum score	-AQ	Frequency	score
0	n=100	3 (3.0%)	2.60	n=100	1 (1.0%)	2.57
1		7 (7.0%)			14 (14.0%)	
2		17 (17.0%)			12 (12.0 %)	
3		73 (73.0%)			73 (73.0 %)	
	Sigmoid - M	Frequency	score	Sigmoid-AQ	Frequency	score
0	n=100	7 (7.0%)	2.33	n=100	4 (4.0%)	2.27
1		12 (12.0%)			24 (24.0%)	
2		22 (22.0%)			13 (13.0%)	
3		59 (59.0%)			59 (59.0%)	
	Descending - M	Frequency	score	Descending-AQ	Frequency	score
0	n=100	5 (5.0%)	2.37	n=100	3 (3.0%)	2.18
1		13 (13.0%)			30 (30.0%)	
2		22 (22.0%)			13 (13.0%)	
3		60 (60.0%)			54 (54.0%)	
MÉDIUM SCORE - LEFT SEGMENT		2.43				2.34
	Transverse - M	Frequency	score	Transverse-AQ	Frequency	score
0	n=100	8 (8.0%)	2.23	n=100	7 (7.0%)	2.1
1		14 (14.0%)			29 (29.0%)	
2		25 (25.0%)			13 (13.0%)	
3		53 (53.0%)			51 (51.0%)	
MÉDIUM SCORE - TRANSVERSE SEGMENT		2.23				2.10
	Ascending - M	Frequency	score	Ascending-AQ	Frequency	score
0	n=100	13 (13.0%)	2.11	n=100	16 (16.0%)	1.74
1		14 (14.0%)			33 (33.0%)	
2		22 (22.0%)			12 (12.0%)	
3		51 (51.0%)			39 (39.0%)	
	Cecum - M	Frequency	score	Cecum-AQ	Frequency	score
0	n=100	17 (17.0%)	1.91	n=100	29 (29.0%)	1.29
1		15 (15.0%)			36 (36.0%)	
2		28 (28.0%)			12 (12.0%)	
3		40 (40.0%)			23 (23.0%)	
	Íleum - M	Frequency	score	Íleum-AQ	Frequency	score
0	n=100	13 (13.0%)	2.26	n=100	17 (17.0%)	1.90
1		6 (6.0%)			24 (24.0%)	
2		23 (23.0%)			11 (11.0%)	
3		58 (58.0%)			48 (48.0%)	
MÉDIUM SCORE - RIGHT SEGMENT		2.09				1.64
FINAL SCORE			6.75			6.10
p			>0.05			>0.05

**FIGURE 1:**
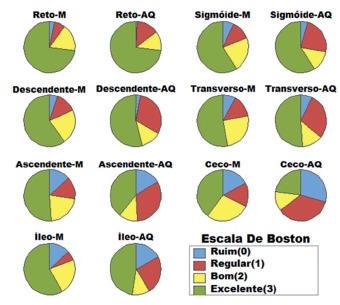
Aquanet EC-2000® (AQ) vs. Manitol (M)

**FIGURE 2:**
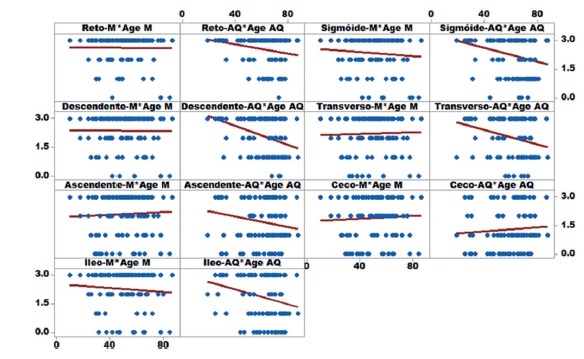
Regression of “age” in “quality of preparation**”**

Statistical analysis applied to different regions of the colon, for both procedures, the proportions observed agreed with the expected (3-excellent). Therefore, the data may be considered non-additive (no bias), i.e., intestinal preparation results using AQ were similar to the results of Mannitol (Figure 1). Furthermore, the regression and residue tests showed that there was autocorrelation (interdependence) between the continuous predictor (age) and the predictors response (quality of the preparation) for the AQ group, with p <0.05, showing that the predictor age influenced the result (Figure 2). The same was not observed in group M. Already the continuous predictor “gender” did not interfere in the predictor response (quality of the preparation, Figure 2).

## DISCUSSION

The present study showed that the AQ process was as effective as Mannitol, based on the Boston Scale score. This device was also shown to be superior to or equal to all the intestinal cleansing procedures found in the literature, both with the use of laxatives (with or without dose separation) and with other cleaning devices^1^ ^,^ ^3^.

Most patients felt more comfortable for intestinal cleansing with AQ, without the adverse effects that occur with laxatives. The unpleasant taste and ingested volume of Mannitol was also taken into account. Also, the new equipment made possible an improvement in the preparation quality. This differentiates it substantially from other procedures found throughout the world. However, AQ has some drawbacks such as cost of deployment and the need for more equipment for multiple exams.

The results obtained with Mannitol are very similar to those presented in the literature^8,9,18^ with a maximum score of 9 by the Boston Scale and p<0.05 for a large number of patients. However, it may present disadvantages of greater patient discomfort and undesirable symptoms. Another difference found between the two methods was obtaining a more comprehensive diagnostic framework for colonoscopy through AQ.

Routine use of colonoscopy for the screening and prevention of colorectal cancer is considered one of the most successful public health projects worldwide^10^ ^,^ ^13^ ^,^ ^16^. Easy acceptance is due to three main factors: first, the technical suitability and evolution of the devices and the safety of the exam; second, to the practical development of the examiner’s skills; third, to the magnificence of the image revealing broad access to the fine features of the mucosa, with comprehensive criteria for diagnosis^1^ ^,^ ^15^ ^,^ ^16^ ^,^ ^19^ ^,^ ^22^.

Thus, adequate preparation has become the most sensitive part of colonoscopy, which is why the present study is under discussion, that is, in search of a fast, efficient, cheap and safe method of preparation^3^ ^,^ ^4^ ^,^ ^8^ ^,^ ^11^ ^,^ ^20^. In the last 40 years, among the various formulas (mechanical and pharmacological) with different associations of laxative drugs, it has been possible to highlight three products that were world references. The solution of 10% Mannitol, solutions of polyethylene glycol and sodium phosphate^4^ ^,^ ^21^.

The questions of these procedures are based on the security that should determine their indiscriminate uses to provide the best preparation conditions. Thus, there is an impasse: Manitol, worldwide banned, continues to be indicated in Brazil, without causing problems, in a dosage regimen different from what was used in the past and which may have influenced the accidents^8^ ^,^ ^17^. On the other hand, the pharmaceutical industry did not succeed in popularizing polyethylene glycol and sodium phosphate in Brazil^4^ ^,^ ^16^ ^,^ ^22^.

In another study with Mannitol, as a comparative example of the present study, patients were randomly divided into four groups. Group A consumed clear liquid diet after lunch the day before the colonoscopy, followed by overnight fasting. Group B, however, received 250 ml of 20% Mannitol and 1 l of 0.9% saline orally at 5 h on the day of the procedure. Group C, the same regimen was done at 20 h the previous day and at 5 h on the day of the examination, and in group D, in addition to group C, 20 ml of simethicone was taken orally 30 min prior to the examination. As a result, preparation of the gut in group D was significantly better than for the other regimens for general bowel cleansing, and showed improvement of general cleansing of the distal small intestine when compared to 10 h of fasting overnight.

Despite the similarity of intestinal preparation with other retrograde methods, AQ is the only equipment currently available that works with pressure and gravity method^1^ ^,^ ^3^ ^,^ ^12^ ^,^ ^15^. It has advantages over others because it is the only one that works with pressure and gravity system, increasing the efficiency of intestinal lavage. In addition, it is a retrograde method of preparation, and there is no need for oral ingestion of laxatives, thus avoiding intolerance to the preparation.

## CONCLUSION

The Aquanet was shown to be as effective as Mannitol for cleaning the three regions of the colon, as well as not causing damage to the intestinal mucosa and better acceptance by the patient.
